# Temperature- and Salt-Resistant Micro-Crosslinked Polyampholyte Gel as Fluid-Loss Additive for Water-Based Drilling Fluids

**DOI:** 10.3390/gels8050289

**Published:** 2022-05-06

**Authors:** Jian Li, Jinsheng Sun, Kaihe Lv, Yuxi Ji, Jingping Liu, Xianbin Huang, Yingrui Bai, Jintang Wang, Jiafeng Jin, Shenglong Shi

**Affiliations:** 1 School of Petroleum Engineering, China University of Petroleum (East China), Qingdao 266580, China; cuplijian@sina.com (J.L.); lvkaihe@upc.edu.cn (K.L.); liujingping20@126.com (J.L.); 20170092@upc.edu.cn (X.H.); smart-byron@163.com (Y.B.); wangjintang@upc.edu.cn (J.W.); jjf5211314@126.com (J.J.); essenssl@163.com (S.S.); 2 CNPC Engineering Technology R&D Company Limited, Beijing 102206, China; 3 Inspection and Testing Center, Huabei Oil Field Company, PetroChina, Renqiu 062552, China; jiyuxi2012@126.com

**Keywords:** water-based drilling fluids, fluid-loss additive, temperature- and salt-resistant, polyampholyte gel, micro-crosslinking

## Abstract

With increasing global energy consumption, oil/gas drilling has gradually expanded from conventional shallow reservoirs to deep and ultra-deep reservoirs. However, the harsh geological features including high temperature and high salinity in ultra-deep reservoirs have become a critical challenge faced by water-based drilling fluids (WDFs), which seriously deteriorate the rheology and fluid loss properties, causing drilling accidents, such as wellbore instability and formation collapse. In this study, a novel temperature- and salt-resistant micro-crosslinked polyampholyte gel was synthesized using *N*,*N*-dimethylacrylamide, diallyldimethyl ammonium chloride, 2-acrylamido-2-methylpropanesulfonic acid, maleic anhydride and chemical crosslinking agent triallylamine through free radical copolymerization. Due to the synergistic effect of covalent micro-crosslinking and the reverse polyelectrolyte effect of amphoteric polymers, the copolymer-based drilling fluids exhibit outstanding rheological and filtration properties even after aging at high temperatures (up to 200 °C) and high salinity (saturated salt) environments. In addition, the zeta potential and particle size distribution of copolymer-based drilling fluids further confirmed that the copolymer can greatly improve the stability of the base fluid suspension, which is important for reducing the fluid-loss volume of WDFs. Therefore, this work will point out a new direction for the development of temperature- and salt-resistant drilling fluid treatment agents.

## 1. Introduction

Drilling fluid or mud, known as the “blood” of drilling engineering, plays vital roles in entire well-drilling operations, such as cleaning the borehole, suspending and carrying drill cuttings, balancing the pressure between the formation and the wellbore, cooling and lubricating the drill bit, etc. [1,2,3,4,5]. Generally speaking, drilling fluids can be divided into water-based drilling fluids (WDFs), oil-based drilling fluids (ODFs), gas-based drilling fluids, etc. [6]. WDFs are a multi-phase dispersion system mainly composed of water, bentonite (BT) and various functional additives (e.g., density modifiers, rheology modifiers, fluid loss additives, inhibitors, plugging agents), which is more environmentally friendly and cheaper than others, thus widely used in drilling engineering [7,8]. At present, with the rapid growth of global petroleum energy demand and the improvement of the technical level of the petroleum industry, oil/gas drilling has begun to extend from medium-shallow reservoirs to deep and ultra-deep reservoirs [9,10,11]. However, the complex geological conditions of deep and ultra-deep wells with high temperature, high salinity and high aquatic hardness seriously affect the performance of WDFs, such as the weakening of the hydration and dispersion of bentonite particles, and the reduction of the effectiveness of functional additives, which results in a series of deterioration of rheology, fluid loss, and sedimentation stability [12,13,14,15]. Therefore, WDFs that can withstand harsh conditions urgently need to be developed, which is of great significance for petroleum engineers to drill into deep oil/ gas resources.

As the main additive of WDFs, fluid loss control additives with excellent temperature- and salt-resistance performance are very important in controlling the fluid loss of drilling fluid and stabilizing the wellbore, having attracted attention from academia and industry [16,17,18]. The fluid loss control agent adsorbed on the surface of the bentonite would prevent the aggregation of bentonite particles and control the particle size distribution of the drilling fluid, thus forming a thin and tough mud cake on the wellbore and reducing the water loss [19,20]. Currently, commonly used fluid loss additives can be divided into two categories according to their sources: natural modified materials and synthetic polymers. In order to improve the temperature resistance and fluid loss reduction ability, a series of natural materials (e.g., cellulose, starch, lignin, xanthan gum) have been extensively researched and applied to WDFs due to their wide range of sources, economic and good environmental performance [21,22,23,24,25]. For example, Li enhanced the rheological and filtration performances of low-solid-content BT-WDFs with nanocellulose [6]. Recently, an environmentally friendly fluid-loss additive based on enzymatic lignin nanoparticles was prepared in our group, which had significant effects on heightening viscosity and debasing fluid-loss [24]. Nevertheless, because of the high content of ether bonds as well as the irregular molecular structures of natural materials, the temperature resistance is usually poor (lower than 180 °C), which limits the applicability in deep and ultra-deep well drilling.

As a comparison, synthesized polymer, with the advantages of adjustable functional groups, adjustable group ratio, regular molecular chain structure, and controllable molecular chain length, has been the mainstream development direction of temperature- and salt-resistant fluid loss agents in the past 20 years [26,27]. Generally speaking, the design of synthetic polymers is mainly from the following aspects: introducing rigid groups in the main chain or side chain to improve the rigidity of molecular chains; introducing adsorption groups (hydroxyl, amino, cationic groups, etc.) to enhance the adsorption capacity of the polymer on the bentonite surface; introducing strong hydration groups (carboxyl groups, sulfonic acid groups, etc.) to enhance the hydration and dispersion ability of bentonite; introducing micro-crosslinking and nanotechnology to further enhance the temperature- and salt-resistance of the polymer. A series of fluid loss additives based on acrylamide (AM) monomers and copolymerized with other functional monomers, such as 2-acrylamide-2-methylpropanesulfonic acid (AMPS), acrylic acid (AA), 4-vinylpyridine (VP), sodium p-styrenesulfonate (SSS), dimethyl diallyl ammonium chloride (DMDAAC) and N-vinylpyrrolidone (NVP), have been synthesized through free radical polymerization [28,29,30]. Although the synthetic polymer fluid loss agent has made sufficient progress in temperature resistance, the polymer molecular chain would curl due to the weakening of electrostatic repulsion in a salt environment containing NaCl, which would cause bentonite particles to agglomerate and lead to a decrease in fluid loss performance. Therefore, synthetic fluid loss reducers that can resist high temperature (up to 200 °C) and resist salinity (saturated salt) are highly desired and still remain a great challenge.

**Figure 1:**
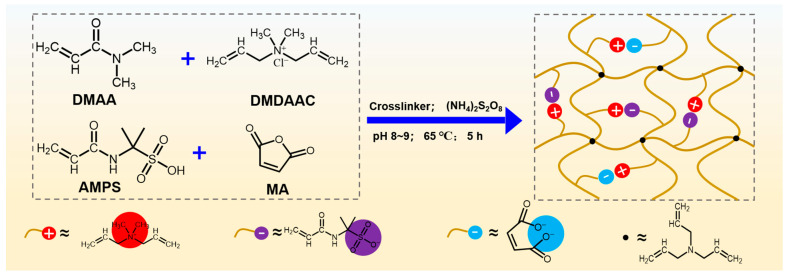
The synthesis procedure and structural formula of DDAM.

Herein, a novel high temperature- and salt-resistant micro-crosslinked polyampholyte gel (i.e., DDAM) was synthesized using *N*,*N*-dimethylacrylamide (DMAA), cationic monomer diallyldimethyl ammonium chloride (DMDAAC), anionic monomer 2-acrylamido-2-methylpropanesulfonic acid (AMPS), maleic anhydride (MA) and chemical crosslinking agent triallylamine through free radical copolymerization, as shown in Figure 1. In this copolymer, DMAA units as the main chain structure enhanced the temperature-resistance of the polymer. DMDAAC as the adsorbing group, enhanced the adsorption capacity of the polymer on the bentonite. AMPS and MA groups, as anionic groups, enhanced the hydration and dispersion of bentonite. Besides, the micro-crosslinked structure changed the condensed structure of the polymer, which restricted the mobility of the polymer segment, thus further improving its temperature- and salt-resistance. Through the optimization of the monomer ratio, the copolymer exhibits excellent fluid loss reduction ability in high temperature and high salt environments. Our strategy may provide new insights into the design of polymers with excellent temperature- and salt-resistance used in deep and ultra-deep well drilling.

## 2. Results and Discussion

### 2.1. Characterization of DDAM

**Figure 2:**
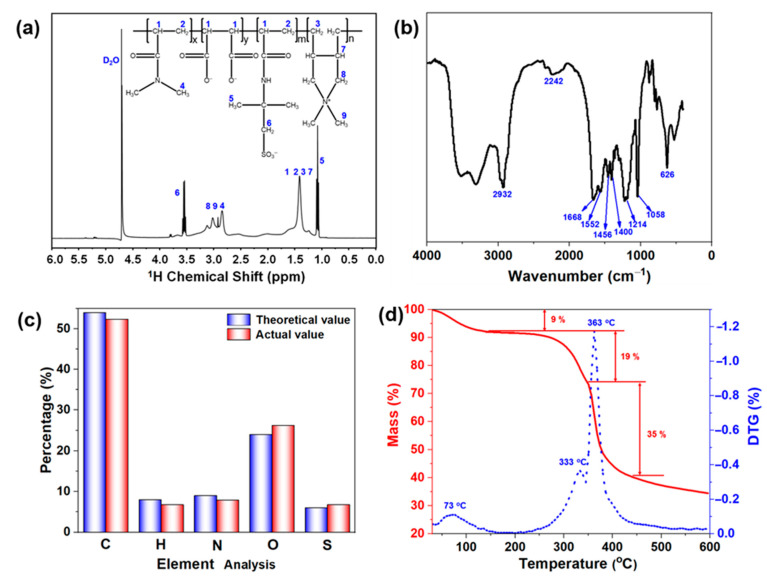
(**a**) ^1^H NMR curves of DDAM. (**b**) FTIR spectrum of DDAM. (**c**) Elemental analysis of DDAM. (**d**) TGA and DTG curves of DDAM.

In order to clarify the structure of DDAM, ^1^H NMR spectroscopy, FTIR and elemental analysis of DDAM were performed. As shown in Figure 2a, the chemical shifts of –CH_2_–CH–CO– (in DMAA or AMPS), –CH–CO– (in MA) and –CH_2_–CH– (in DMDAAC) groups were at the regions of 1.25–1.75 ppm. The CH_3_– proton peaks in DDAM appeared at 3.0 ppm. The C–2(CH_3_)– and –CH_2_–SO_3_– in AMPS could be observed at 1.0 ppm and 3.1 ppm, respectively. Peak (3.0–3.3 ppm) should be attributed to the CH_3_–N^+^ and –CH_2_–N^+^ in DMDAAC. As a result, the above characteristic peaks of atomic groups demonstrated the successful synthesis of the copolymer. In addition, the FTIR spectra of DDAM was shown in Figure 2b, and the peak at a wavelength of 2932 cm^−1^ was caused by the asymmetric stretching vibration of CH_2_ in the main chain of the copolymer. The peaks at wavelengths of 1668 and 1456 cm^−1^ were caused by the carbonyl C=O in the amide group and the stretching vibrational peak of –CN– in DMAA, respectively. The broad peak at a wavelength of 2242 cm^−1^ was attributed to the stretching vibration peaks of the carbonyl C=O in MA. The peaks at 1552 and 1400 cm^−1^ were attributed to the -NH bond on the amino group and the vibrational absorption peak of the five-membered heterocyclic –CN– in DMDAAC, respectively. The peak at 626 cm^−1^ corresponded to the absorption peak of –CS in AMPS while the peaks at 1214 and 1058 cm^−1^ were attributed to the stretching vibrations of the sulfonic acid groups in AMPS. Besides, the characteristic peaks of unsaturated olefin bonds in DMAA, MA, AMPS and DMDAAC monomers did not appear on the FTIR spectral, indicating that the monomer reaction was complete. Moreover, as shown in Figure 2c, the elemental analysis (C, H, N, O, S) of the copolymer synthesized with the optimal monomer molar ratio of DMAA, MA, AMPS, and DMDAAC (5:3:2:2) shows that the theoretical value of the element content in the copolymer was consistent with the measured value, indicating the resulting product was the target product.

Since the polymer additives are essential for maintaining the function of drilling fluids at high temperatures, excellent thermal stability is an indispensable performance for drilling fluid treatment agents. To demonstrate the thermal stability of DDAM copolymer, thermogravimetric analysis (TGA) and differential thermogravimetric (DTG) curves of DDAM were performed in a N_2_ atmosphere, and the mass loss of the polymer could be divided into four stages (Figure 2d). The first stage ranged from 25 °C to 220 °C and the peak occurred at 73 °C, with a mass loss of 9%, which was mainly attributed to the evaporation of water molecules adsorbed on the copolymer. In the second stage with the temperature ranging from 220 °C to 350 °C and the peak occurring at 333 °C, the mass loss was about 19%, which mainly corresponds to the decomposition of the amide group of the copolymer. The third stage was 350 °C to 450 °C with the peak at 363 °C, where the sulfonic groups and C-C bonds in the main chains of polymer began to degrade, resulting in a weight loss of 35%. When the temperature exceeded 450 °C, the polymer gradually carbonized, thus causing mass loss. Therefore, DDAM copolymer exhibited excellent thermal stability and had great potential for application in high temperature resistant drilling fluids.

### 2.2. Rheological Properties of DDAM Solutions and DDAM-Based Drilling Fluid

**Figure 3:**
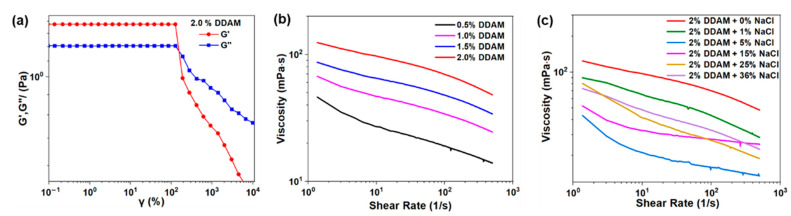
(**a**) Viscoelastic behavior of DDAM solution (**b**) Viscosity curves of DDAM with different concentration (**c**) Viscosity curves of 2 wt% DDAM in different NaCl concentration.

Since the rheology of the drilling fluid is essential for carrying and suspending cuttings, as well as maintaining the stabilization of the wellbore, the rheological properties of the DDAM solutions and DDAM-based drilling fluid were analyzed, respectively. As shown in Figure 3a, when the strain is less than 100%, the elastic modulus G′ and viscous modulus G″ remain almost unchanged, indicating that DDAM solutions have a wide linear viscoelastic region. Additionally, as shown in Figure 3b, the viscosity of the DDAM solution gradually decreased with the increase in shear rate, showing shear thinning behavior, which was beneficial to the drilling fluid to suspend the cuttings at a low shear rate and break the rock at high shear rate. Moreover, as shown in Figure 3c, when the concentration of the salt increased, the viscosity of the DDAM solution decreased rapidly and then slowly increased. This was because a small amount of sodium ions would have a dehydration effect on the anionic groups that were not bound by electrostatic attraction, causing the viscosity of the polyampholyte solution to decrease rapidly. However, when the sodium ion reached a certain value, the metal sodium ion with high charge density would weak the electrostatic force between the anionic and cationic groups suspended on the polymer, thus the polymer chain gradually transited from the curled state to the stretched state, with the viscosity of the solution no longer drop or even increase.

**Figure 4:**
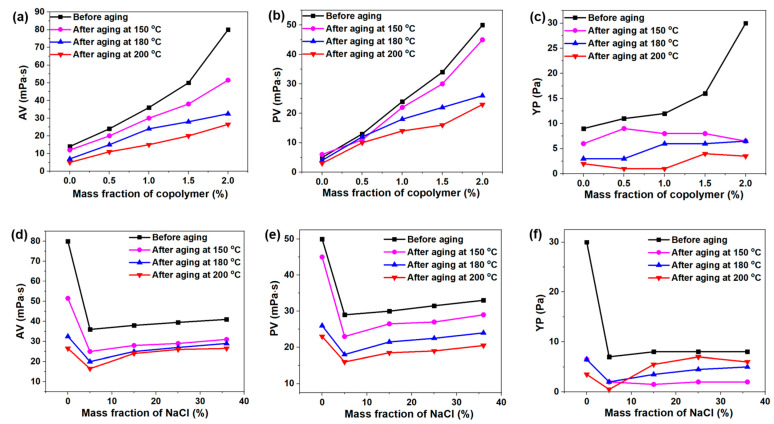
Rheological properties of DDAM-based drilling fluid with different contents before aging and after aging at 150 °C, 180 °C and 200 °C for 16 h: (**a**) AV; (**b**) PV; (**c**) YP. Rheological properties of 2 wt% DDAM-based drilling fluids with different fraction of NaCl before aging and after aging at 150 °C, 180 °C and 200 °C for 16 h: (**d**) AV; (**e**) PV; (**f**) YP.

Further, as shown in Figure 4a–c, the rheological parameters (AV, PV and YP) of the base drilling fluid were relatively low, but gradually increased with the addition of the copolymer. When 2 wt% DDAM was added into the base drilling fluid, the AV increased from 10 to 80 mPa·s (Figure 4a), the PV increased from 4 to 50 mPa·s (Figure 4b), and the YP increased from 8 to 30 (Figure 4c), demonstrating the copolymer has excellent rheological adjustment properties. Generally speaking, when the AV value in WDFs was greater than 80, it would weaken the fluidity of the WDFs, and even lead to drilling accidents, such as stuck drills in severe cases [31]. Therefore, 2 wt% copolymer was the best from the perspective of economy and safety. As we know, the rheological properties of the drilling fluid would become worse after high temperature aging, which results in the deterioration of its performance. This was because the high temperature would lead to a decrease in the adsorption capacity of the polymer on the bentonite, which weaken the hydration and dispersion properties of bentonite. Even so, the AV and PV of the base drilling fluid with 2 wt% DDAM after hot roll at 200 °C for 16 h were higher than the base fluid, indicating the DDAM copolymer has excellent temperature-resistance and can protect the stability of base drilling fluid at high temperature.

Usually, salt would affect the performance of WDFs from the following two aspects: one is the metal cations adsorbed on the surface of the bentonite would compress the electric double layer and decrease the electrostatic repulsion between bentonite, which causes the agglomeration of bentonite; on the other hand, the salt would cause the polymer chain to curl, which weakens the interaction between the polymer and the bentonite, thus increasing the particle size and the fluid loss of the drilling fluid. In order to test the salt pollution resistance of drilling fluid, the influence of electrolytes on the rheological properties of drilling fluid based on 2 wt% DDAM content was studied. As shown in Figure 4d–f, the AV of the basic drilling fluid with the addition of 2 wt% DDAM decreased from 80 to 36 mPa·s, the PV decreased from 50 to 29 mPa·s and the YP decreased from 30 to 7 mPa·s after contamination with 5 wt% NaCl, which confirmed that a small amount of salt would have a serious negative impact on the rheology of the drilling fluid. However, when the salt concentration continued to increase to saturation (36 wt%), the rheological parameters of the drilling fluid gradually increased, with the AV increasing to 41, the PV increasing to 33, and the YP increasing to 8. The reason account for this phenomenon was as follows: when a small amount of salt was added to the drilling fluid, the hydration and dispersion ability of the bentonite would rapidly weaken. Besides, the anionic groups suspended on the polymer do not form an electrostatic connection and would rapidly dehydrate, which further increased the curling of the polymer molecular chain, resulting in a decrease in the viscosity of the drilling fluid. When the salt concentration increased to a certain value, Na^+^ destroyed the electrostatic force between the carboxyl group, sulfonic acid group and quaternary ammonium group, and reduced the degree of folding and curling of the polymer chain, which lead to the increase of polymer hydrodynamic volume, thus increasing the viscosity of the drilling fluid.

### 2.3. Fluid Loss Analysis of DDAM-Based Drilling Fluid

**Figure 5:**
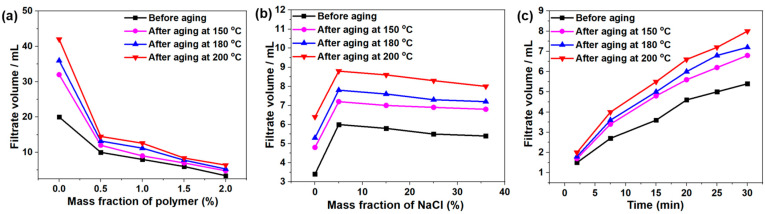
(**a**) API filtration loss of DDAM-based drilling fluid before aging and after aging at 150 °C, 180 °C and 200 °C for different contents. (**b**) API filtration loss of 2 wt% DDAM-based drilling fluids with different fraction of NaCl before aging and after aging at 150 °C, 180 °C and 200 °C. (**c**) API filtration loss of 2 wt% DDAM-based drilling fluids in saturated salt environment before aging and after aging at 150 °C, 180 °C and 200 °C for different time.

Driven by the positive pressure difference between the mud static column pressure and the formation wellbore pressure, the liquid part, as well as solid particles of the drilling fluid, would permeate into the formation through the wellbore cracks, gradually forming a low permeability mud cake on the wellbore, which was called the filtrate loss and wall-building functions of drilling fluid. The fluid loss performance of drilling fluid mainly referred to the fluid loss volume of the drilling fluid and the quality of the formed mud cake, which was very important to maintain the stability of the wellbore. For the evaluation of fluid loss of drilling fluid, the American Petroleum Institute (API) standard states were usually used. It could be seen from Figure 5a that as the DDAM concentration increased, the fluid loss before and after the aging of the drilling fluid gradually decreased. When 2 wt% copolymers were added, the fluid loss of the drilling fluid before aging reduced from 20 mL to 3 mL, and the fluid loss reduced from 42 mL to 6.5 mL after aging at 200 °C, which showed that the DDAM had an excellent high-temperature filtration reduction performance. After the electrolyte invades the drilling fluid system, it often leads to an increase in the fluid loss of the drilling fluid. The relationship between the filtration volume of the drilling fluid containing 2 wt% DDAM and the sodium chloride concentration was shown in Figure 5b. When the concentration of sodium chloride increased to 5 wt%, due to the polyelectrolyte effect, the filtration volumes of drilling fluids before aging and after aging at 200 °C increased from 3.5 to 6 mL and 6.4 to 8.8 mL, respectively. However, when the salt concentration was further increased, the fluid loss of the drilling fluid showed a slowly decreasing trend because of the reverse polyelectrolyte effect. When the concentration of salt increased to 36 wt%, the fluid loss of the drilling fluid before aging was 5.4 mL, and after aging at 200 °C was 8 mL, indicating the copolymer had excellent high temperature and high salt fluid loss reduction performance. Simultaneously, Figure 5c shown the variation curves of filtrate volume of drilling fluid containing 2 wt% copolymers in a saturated salt environment before and after aging with time, which further confirmed the DDAM-based drilling fluids have good filtration loss reduction performance.

### 2.4. Mechanistic Analysis

**Figure 6:**
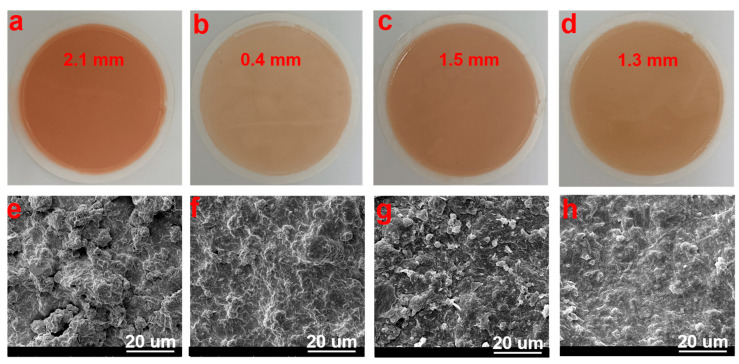
Digital images of mud cakes deposited from different WDFs after aging at 200 °C for 16 h: (**a**) 4% bentonite, (**b**) 4% bentonite + 2 wt% DDAM, (**c**) 4% bentonite + 2 wt% DDAM + 5 wt% NaCl, (**d**) 4% bentonite + 2 wt% DDAM + 36 wt% NaCl. SEM micrographs of dried filter cakes deposited from different WDFs after aging at 200 °C for 16 h: (**e**) 4% bentonite, (**f**) 4% bentonite + 2 wt% DDAM, (**g**) 4% bentonite + 2 wt% DDAM + 5 wt% NaCl, (**h**) 4% bentonite + 2 wt% DDAM + 36 wt% NaCl.

The quality of the mud cake formed in the drilling process not only directly affects the filtration loss of the drilling fluid system but also is closely related to the drilling safety, which occupied important practical significance in drilling engineering. When the fluid loss reducer is dissolved in the base drilling fluid, the polar and cationic groups on the copolymer can be adsorbed on the bentonite surface to prevent the bentonite particles from accumulating and becoming larger. To confirm that DDAD can improve the quality of the mud cake and reduce the filtration loss of drilling fluid, the macro and micromorphology of the mud cake formed by drilling fluid after aging at 200 °C were studied. The mud cake formed by the base slurry aged at 200 °C was rough and with a thickness of 2.1 mm (Figure 6a). This was because the repulsive force between the bentonite particles was weakened after high temperature aging, which caused the bentonite particles to coalesce and increase the average particle size (Figure 6e). However, when 2 wt% DDAM was added to the base slurry, the thickness of the mud cake was reduced to 0.4 mm (Figure 6b), and the surface became denser with only smaller particles (Figure 6f). This indicated that the DDAD copolymer after high temperature aging could still be adsorbed on the surface of bentonite particles, inhibiting the agglomeration of bentonite particles, and improving the quality of the mud cake. Generally, the positive Na^+^ would compress the electric double layer between the bentonite particles, which weakens the hydration and dispersion of the bentonite, resulting in poor quality of the mud cake and increased fluid loss. When 5 wt% NaCl was added to the drilling fluid containing 2 wt% DDAM, the thickness of the mud cake increased to 1.5 mm (Figure 6c), and some agglomerated bentonite particles appeared on the surface (Figure 6g). However, the particle size distribution of the bentonite particles was still relatively suitable, and the stacking between the particles was relatively tight, indicating that the copolymer still had a good adsorption capacity in a salt-containing environment. Interestingly, when the NaCl concentration reached 36 wt%, the polymer chain of DDAD stretched due to the reverse polyelectrolyte effect and the adsorption group could effectively adsorb on the surface of bentonite particles. As a result, the thickness of the mud cake was slightly reduced to 1.3 mm (Figure 6d), and the agglomerated particles on the surface were also reduced (Figure 6h), which demonstrated the DDAD can effectively control the filtration loss of drilling fluid under high temperature and high salinity conditions.

**Figure 7:**
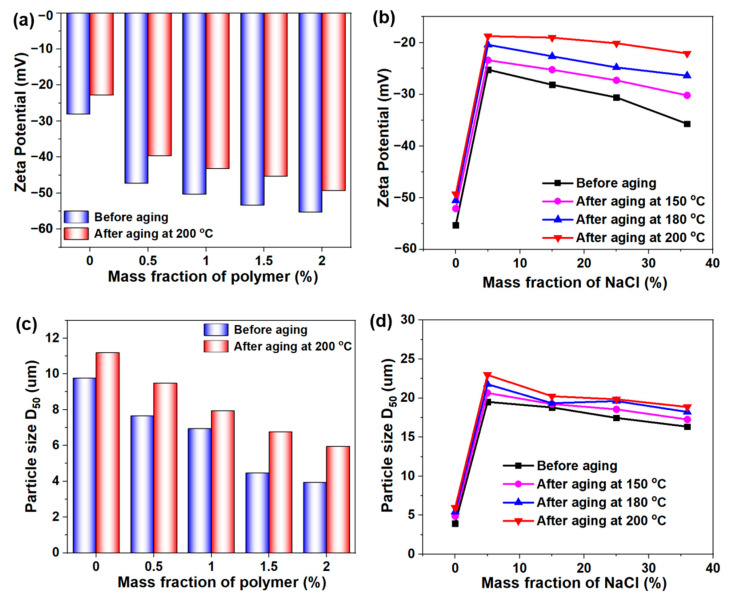
(**a**) Zeta potential curves of DDAM-based drilling fluid before aging and after aging at 200 °C. (**b**) Zeta potential curves of 2 wt% DDAM-based drilling fluid with different fraction of NaCl before aging and after aging at 200 °C. (**c**) Particle size of DDAM-based drilling fluid before aging and after aging at 200 °C. (**d**) Particle size of 2 wt% DDAM-based drilling fluid with different fraction of NaCl before aging and after aging at 200 °C.

Drilling fluid is a colloidal dispersion system composed of bentonite and water. The zeta potential can reflect the repulsive force between colloidal particles, which in turn reflects the colloidal stability of the drilling fluid system. As shown in Figure 7a, with the increase of DDAM copolymer concentration, the zeta potential (absolute value) of the drilling fluid gradually increased, which indicated the DDAM copolymer can effectively be adsorbed on the bentonite particles and enhanced the stability of the dispersion system. As mentioned above, the addition of salt would compress the electric double layer of the bentonite, thus causing a drop in zeta potential. After the addition of 5 wt% salt, the zeta potential of the drilling fluid dropped sharply, but it gradually recovered when the concentration of salt was increased to 36 wt% due to the reverse polyelectrolyte effect (Figure 7b). In addition, the adsorption capacity of the DDAM copolymer on the bentonite was weakened at high temperatures, and the zeta potential of the drilling fluid decreased with increasing aging temperature. In order to form a thin and tough mud cake and reduce the filtration loss of the drilling fluid, it was required that there should be a certain number of large-sized bentonite particles as bridging particles and a certain number of small-sized bentonite particles as filler particles in the drilling fluid system, which means that the drilling fluid system must have a reasonable particle size distribution. The effects of copolymer content, temperature and salt concentration on the median particle size (D50) of drilling fluid were analyzed by a laser particle size analyzer. As shown in Figure 7c, the median particle size of the drilling fluid gradually decreased with the increase of the DDAM concentration because the polymer could effectively be adsorbed on the bentonite particles and inhibited the aggregation of the bentonite particles. When 5 wt% NaCl was added into the drilling fluid, Na^+^ would weak the hydration and dispersion of the bentonite particles, causing the coalescence of the bentonite particles and an increase in the median particle size. However, with a further increase in NaCl concentration, the particle size of the drilling fluid tends to decrease slightly (Figure 7d). This was because the reverse polyelectrolyte effect of the DDAM benefits the adsorption of the bentonite in the high-salt environment, thus the bentonite particles maintain a certain degree of dispersion.

## 3. Conclusions

In this work, a novel high temperature- and salt-resistant micro-crosslinked polyampholyte gel filtration reducer (i.e., DDAM) was synthesized using DMAA, DMDAAC, AMPS, MA and chemical crosslinking agent triallylamine through aqueous free-radical copolymerization. TGA analysis demonstrated the DDAM copolymer exhibited excellent thermal stability and had great potential for application in high temperature resistant drilling fluids. Meanwhile, due to the reverse polyelectrolyte effect, DDAM still shows the ability to increase viscosity in high saltwater environments. As a result, when 2 wt% DDAM was added to the base drilling fluid, the DDAM-based drilling fluids exhibited outstanding rheological and filtration properties (FL_API_ < 10 mL) even after aging at high temperatures (up to 200 °C) and high salinity (saturated salt) environments. Moreover, the zeta potential and particle size distribution of DDAM-based drilling fluids further confirmed the enhanced rheological and filtration properties could be attributed to the presence of positive and negative functional groups which could effectively be adsorbed on the bentonite particles and inhibit the aggregation of the bentonite particles. Overall, this work can promote the application of micro-crosslinked polyampholyte gel as a fluid-loss additive for WDFs under extremely high temperature and high salinity conditions.

## 4. Materials and Methods

### 4.1. Materials

Monomers *N*,*N*-dimethylacrylamide (DMAA, 98 wt%), diallyldimethyl ammonium chloride (DMDAAC, 90% solution), 2-acrylamido-2-methylpropanesulfonic acid (AMPS, 99 wt%), maleic anhydride (MA, 98 wt%) were purchased from Sigma-Aldrich Corporation (Shanghai, China). Initiator ammonium persulfate (APS, 99 wt%), chemical crosslinking agent triallylamine (99 wt%), and sodium hydroxide (NaOH, 99 wt%) were obtained from J&K Chemical, Ltd. (Beijing, China). Bentonite (sodium form) was from Weifang, Shandong. All reagents were used as received without further purification unless specified.

### 4.2. Synthesis of DDAM

First, 12.38 g DMAA, 7.35 g MA, 10.35 g AMPS, 8.05 g DMDAAC and 0.20 g chemical cross-linking agent triallylamine (0.5 wt% of the total monomer mass) were dissolved in deionized water (pH adjusted to 8~9 with sodium hydroxide), and the total mass fraction of reactive monomers was adjusted to 30 wt%. Secondly, the above solution was put into a three-necked flask, stirred at high speed for 30 min under the protection of nitrogen, and heated to the reaction temperature (60 °C). Finally, 0.08 g ammonium persulfate initiator (0.2 wt% of the total monomer mass) was added to initiate the polymerization reaction for 8 h. After the reaction, the solution was washed with a mixed solution of absolute ethanol and acetone, precipitated, and filtered to obtain a micro-crosslinked polyampholyte gel fluid loss control agent (DDAM).

### 4.3. Methods

#### 4.3.1. DDAM Characterization

NMR spectra of DDAM were acquired using an NMR spectrometer (Bruker AVANCE III NMR) with deuterated water (D_2_O) as the solvent. Fourier transform infrared (FTIR) spectrometry (A225/Q Platinum ATR) was also conducted to confirm the structure of DDAM. The elemental composition of DDAM was analyzed by the Elementar Vario EL instrument. The thermal property of DDAM was evaluated by the thermogravimetric analyzer (Mettler Toledo Co., Zurich, Switzerland) under nitrogen purging and a heating rate of 10 °C/min.

#### 4.3.2. Preparation of Water-Based Drilling Fluids (WDFs)

For base drilling fluid preparation, 16 g bentonite was dispersed uniformly in 400 mL of water and stirred at 5000 rpm for 2 h, followed by static standing at room temperature for 24 h. For copolymer-based drilling fluid, a certain amount of copolymer (DDAM) was dissolved in 400 mL of base drilling fluid with 8000 rpm stirring for 20 min. For salt contamination, different mass fractions of sodium chloride were slowly added into the DDAM-based drilling fluid at 5000 rpm stirring for 20 min. In order to test the temperature- and salt-resistance of the WDFs, the above-mentioned salt-contaminated DDAM-based drilling fluid was added to a roller oven (Fann Instrument Company) and aged at different temperatures for further trials.

#### 4.3.3. American Petroleum Institute (API) Fluid-Loss Test

The API filtrate performance test was measured by using a ZNZ-D3 medium pressure filtration loss apparatus (Qingdao Haitongda Special Instrument Co., Ltd., Qingdao, China). The volume of a certain volume of fluid filtered through filter paper (9 mm diameter and 2.7 μm pore size) for 30 min under a pressure of 0.7 MPa was the API filtrate volume.

#### 4.3.4. Rheology Analysis

The rheology of the DDAM solution was measured by a rotary rheometer (TA, DHR-2). The elastic and viscous moduli of DDAM solution as a function of strain were obtained by the oscillatory shear mode (γ: 0.1~10,000%, f: 1 Hz). The viscosity of the DDAM solution was measured under the shear rate from 1 to 500 s^−1^ and the frequency was 1 Hz. In addition, the rheology of DDAM-based drilling fluid was measured according to the American Petroleum Institute (API) standards. Firstly, test the dial reading Ф_600_ (600 r/min) and Ф_300_ (300 r/min) by a six-speed rotating viscometer (model 35A viscometer, Fann Instrument Company, Houston, TX, USA). Thereafter, calculate the apparent viscosity (AV), plastic viscosity (PV), and yield point (YP) from the viscometer test data:

Apparent Viscosity (AV) = 0.5Ф_600_ (mPa·s)

Plastic Viscosity (PV) = Ф_600_ − Ф_300_ (mPa·s)

Yield Point (YP) = Ф_300_ − 0.5Ф_600_ (Pa)

## Data Availability

We don’t have any data.
